# Novel Cervical Endoscopic Unilateral Laminoforaminotomy for Bilateral Decompression in Cervical Spondylosis Myeloradiculopathy: A Technical Note and Clinical Results

**DOI:** 10.3390/jcm13071910

**Published:** 2024-03-26

**Authors:** Kai-Ting Chien, Yu-Cheng Chen, Ting-Kuo Chang, Yueh-Ching Liu, Lei-Po Chen, Yu-Ching Huang, Yan-Shiang Lian, Jian-You Li

**Affiliations:** 1 Department of Orthopaedic Surgery, MacKay Memorial Hospital, Taipei 104217, Taiwan; ktchien.hs11@nycu.edu.tw (K.-T.C.);; 2 Institute of Applied Arts, National Yang Ming Chiao Tung University, Hsinchu 30010, Taiwan; 3 Department of Medical Education, MacKay Memorial Hospital, Taipei 104217, Taiwan; 4 Department of Medicine, Mackay Medical College, No.46, Sec. 3, Zhongzheng Rd., Sanzhi Dist., New Taipei City 252, Taiwan

**Keywords:** cervical spondylosis, cervical endoscope, Laminoforaminotomy, myelopathy, radiculopathy

## Abstract

**Background**: This study investigates the efficacy of the Cervical Endoscopic Unilateral Laminoforaminotomy for Bilateral Decompression (CE-ULFBD) technique in treating cervical myeloradiculopathy, primarily caused by degenerative spondylosis. Traditionally managed through multisegmental anterior cervical discectomy and fusion (ACDF) or laminoplasty combined with foraminotomy, this condition has recently experienced a promising shift towards minimally invasive approaches, particularly endoscopic spinal decompression. While empirical evidence is still emerging, these techniques show potential for effective treatment. **Method**: The objective was to evaluate the outcomes of CE-ULFBD in achieving single or multilevel bilateral foraminal and central decompression, emphasizing the reduction of injury to posterior cervical muscles and the associated postoperative neck soreness common in conventional procedures. This paper delineates the surgical procedures involved in CE-ULFBD and presents the clinical outcomes of nine patients diagnosed with myeloradiculopathy due to severe cervical stenosis. **Result**: Assessments were conducted using the Visual Analogue Scale (VAS) for neck and arm pain and the Modified Japanese Orthopaedic Association scale (mJOA) for the activity measurement of daily living. Results indicated a considerable decrease in pain levels according to the VAS, coupled with significant improvements in functional capacities as measured by the mJOA scale. Additionally, no major postoperative complications were noted during the follow-up period. **Conclusion**: The study concludes that CE-ULFBD is a safe and effective approach for the treatment of cervical myeloradiculopathy resulting from severe cervical stenosis, offering a viable and less invasive alternative to traditional decompressive surgeries.

## 1. Introduction

Cervical spondylosis (CS) is an age-related disorder due to osseocartilaginous changes that affect approximately 85% of individuals older than 60 years old [1]. It can be classified according to clinical presentations into axial neck pain, radiculopathy, myelopathy, or myeloradiculopathy [2]. Myeloradiculopathy is a combination of spinal cord and nerve root involvement that can be caused by a variety of factors, such as ligamentum flavum hypertrophy, ossification of the Posterior Longitudinal Ligament, herniated discs, foraminal spurs, spinal cord tumors, infarction, and traumatic events [3,4,5]. Patients with myeloradiculopathy may experience symptoms of dermatomal radicular pain and numbness, as well as weakness in the extremities, gait disturbances, and urinary problems associated with myelopathy [6,7]. These severe and debilitating symptoms can greatly impact an individual’s quality of life and make it challenging to complete ordinary tasks.

When conservative treatments fail to relieve symptoms caused by nerve compression, surgical options such as Anterior Cervical Discectomy and Fusion (ACDF) and Posterior Open Laminoplasty with Foraminotomy are available to prevent worsening neurological function in patients with myeloradiculopathy [8,9]. While ACDF has been a gold standard since the 1950s, its anterior approach sometimes results in complications like dysphagia, hoarseness, and issues related to fusion. Alternatively, Posterior Open Laminoplasty with Foraminotomy offers a dynamic, preservation-oriented approach to access the spine, minimizing ventral injury. However, it also poses its challenges, including poor outcomes for segmental kyphosis, risk of facet joint instability, and postoperative axial neck pain.

To reduce these complications and improve patient satisfaction, endoscopic techniques for the cervical spine have been developed to achieve sufficient nerve decompression with decreased postoperative morbidity [10]. Endoscopic posterior cervical spinal decompression surgery is performed using specialized instruments, including endoscopic forceps, radiofrequency probes, and burrs. These tools are employed to excise degenerated or compressive tissues and vaporize intervertebral disc material and ligaments, thereby alleviating pressure on the nerve roots and spinal cord [11]. While these techniques have demonstrated clinical outcomes comparable to traditional open surgery, their application has primarily been studied and documented in patients with either cervical radiculopathy or myelopathy separately. However, there is a notable gap in the literature regarding their use in treating cervical myeloradiculopathy, a condition that combines both radiculopathy and myelopathy. To bridge this gap, we present a series of cases with myeloradiculopathy. In these cases, we explore the surgical technique and outcomes of Cervical Endoscopic Unilateral Laminoforaminotomy for Bilateral Decompression (CE-ULFBD), a procedure that involves a single-sided approach for bilateral foraminotomy and central decompression, a novel application in this context.

## 2. Methods

Between 2020 and 2022, we conducted a retrospective review of nine patients, four men, and five women, with an average age of 69.44 ± 5.15 years (range: 62–75 years) (Table 1), who had been diagnosed with myeloradiculopathy and underwent CE-ULFBD at our institution. After obtaining informed consent and approval from our institutional ethics committee, we examined demographic data, operative details, preoperative images, and patient-reported outcomes. Participants were selected based on a diagnosis of bilateral radiculopathy due to lateral or intraforaminal disc herniation, foraminal osteophytes, or myelopathy associated with ligamentum flavum hypertrophy or ossification, degenerative spurs, or bulging discs. Patients had engaged in over three months of conservative therapies without improvement. Exclusion criteria included cervical instability, kyphotic deformity, OPLL, surgical intolerance, infection, and spinal cord tumors.

Clinical and imaging assessments were used to diagnose cervical radiculopathy and myelopathy, with radicular symptoms often presenting as bilateral arm pain, numbness, tingling in a dermatomal pattern, and specific muscle group weakness. Physical exams showed positive results in Spurling’s, axial traction, and Arm Squeeze tests, while myelopathy was indicated by lower extremity weakness, gait disturbances, fine motor skill challenges, and positive Romberg and Hoffmann’s tests. Preoperative pain levels were quantified using the VAS for neck and arm pain, with mean scores of 7.29 ± 0.73 and 6 ± 0.82, respectively. The mJOA score, indicative of the activity of daily living, averaged 14.71 ± 0.8 across the cohort. The mJOA (modified Japanese Orthopaedic Association) score ranges from 0 to 18, where a higher score indicates better neurological function [12].

## 3. Statement

The study adhered to the Declaration of Helsinki’s principles and received approval from our institution’s ethics committee (IRB approval number 23MMHIS171e). Written and verbal informed consent was obtained from all participants. The reporting of this study conformed to the PROCESS (Preferred Reporting of Case Series in Surgery) guidelines.

## 4. Surgical Technique (Video)

**(a)**  **Anesthesia, Position, and Equipment Setting**

**Figure 1:**
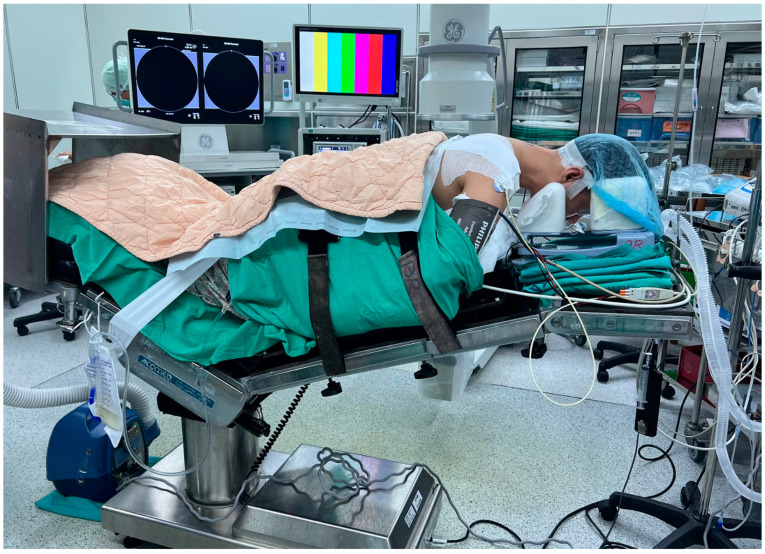
This figure illustrates the patient positioning for the CE-ULFBD surgery, with the patient in the prone position. The surgeon stands on the side where radiculopathy is more pronounced. The C-arm and the endoscope monitor are positioned on the contralateral side. Notably, this surgical procedure incorporates MEP (Motor Evoked Potential) and SSEP (Somatosensory Evoked Potential) for enhanced neural monitoring.

All patients underwent general anesthesia and were placed prone with Mayfield tongs on a Jackson table. All patients had intraoperative neuromonitoring, including motor-evoked potentials (MEP) and somatosensory-evoked potentials (SSEP) (Figure 1). The patient’s hair was shaved up to the occipital inferior margin, and the head was positioned slightly forward-flexed to open the interlaminar space. The video monitor for the endoscope and the fluoroscopy C-arm were positioned on the opposite side of the patient’s body where their symptoms were located. Normal saline was utilized for irrigation, with precautions taken to avoid the use of pumping, as it could elevate intracranial pressure due to the close proximity of the irrigation channel to the brain. The saline bags were hung at a height of 0.5 m above the patient’s horizontal level, ensuring sufficient fluid flow for clear endoscopic vision. For every 1.5 L of normal saline, 1 mg of epinephrine is added to aid in hemostasis. The surgical team maintained open communication with the anesthesiologist and informed the patient of a systolic blood pressure goal of <110 mmHg to control bleeding efficiently. Two units of transamin were administered intravenously as a push, and an additional two units were delivered via IV drip prior to incision.

**(b)**  **Wound Incision and Endoscope Placement**

**Figure 2:**
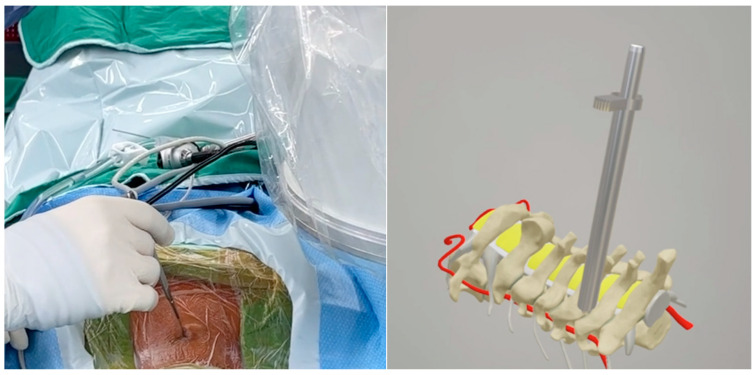
The incision was made near the midline, close to the spinous process; the level was subsequently confirmed through lateral fluoroscopic imaging.

**Figure 3:**
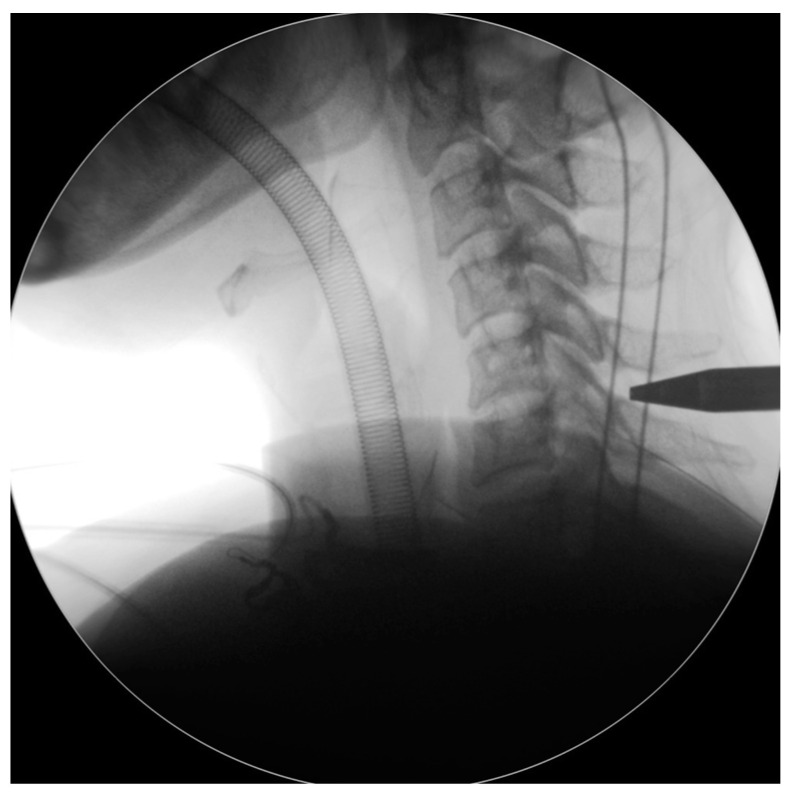
Check the intraoperative fluoroscopic images to ensure the dilator tip landed on the correct level.

The endoscope was inserted on the dominant side with more intense radiculopathy or myelopathy. A 1-cm skin incision was made near the midline, adjacent to the spinous process, with the precise location confirmed by utilizing both anterior-posterior and lateral fluoroscopic imaging. (Figure 2). We then carefully dissected the fascia through mosquito forceps and passed the muscle layer with a blunt tip dilator. After feeling the edges of the lamina at the targeted spinal level, we placed the working sleeve using the dilator. Then, we rechecked the anterior-posterior and lateral fluoroscopic images after removing the dilator and inserting the endoscope through the working sleeve to ensure its tip landed on the correct level (Figure 3). Likewise, we opted for a 15° endoscope with a 10-mm outer diameter and a 7.1-mm working channel (LUSTA endoscopic system, Spinendos, Germany) to facilitate a more versatile approach to bony decompression through this relatively larger scope.

**(c)**  **Decompression**

**Figure 4:**
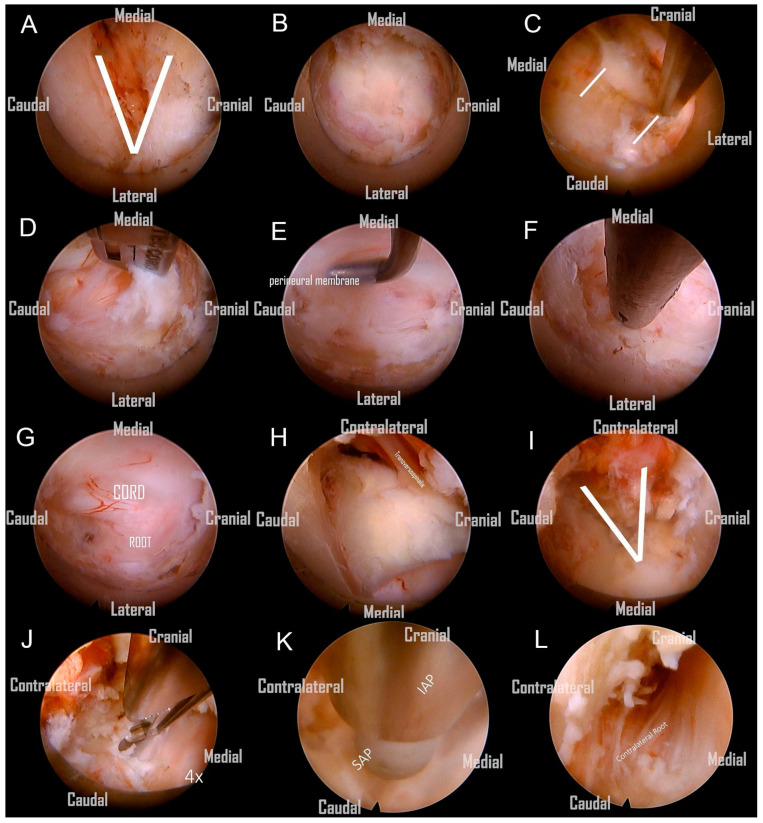
(**A**) Recognize the V-point. (**B**) O-shape foraminotomy. (**C**) Identify the lateral border of the facet to avoid injuring the vertebral artery or causing facet instability. (**D**) Remove the ipsilateral ligamentum flavum piece by piece and expose the ipsilateral nerve root. (**E**) Resect the perineural membrane to expose the nerve root. (**F**) Resect the protrusive degenerative disc. (**G**) Optimal decompression of the ipsilateral spinal cord and nerve root achieved. (**H**) Burr the central lamina and spinal process base for the opposite work. (**I**) Burr the opposite lamina gradually and find the opposite V point. (**J**) Remove the contralateral ligamentum flavum. (**K**) Then, we switched instruments to a standard 30° endoscope with a 6.9-mm outer diameter and a 4.3-mm working channel (FELD scope, Spinendos GmbH, München, Germany). This enabled more thorough contralateral foraminotomy and decompression of the opposite foraminal stenosis. (**L**) The contralateral perineural membrane was excised, thereby exposing the contralateral nerve root.

Following the removal of the connective tissue, the V-point was accurately identified. (Figure 4A). The ‘O-shape foraminotomy’ was performed at the edge of the ‘V-point’ using a diamond burr. Use the side of the burr head to grind the bone, and be careful not to press the burr head downward forcefully. The procedure involved initially grinding at the cranial end, then progressively moving towards the caudal end. This approach is tailored to the anatomical orientation, as some parts of the lamina are overlapped cranially over the caudal end. This gradual approach effectively enlarges the space, subsequently facilitating the convenient use of Kerrison rongeurs. (Figure 4B). Identifying the lateral border of the facet is also critical to avoid the foraminotomy from going too far laterally, as it would injure the vertebral artery or cause facet instability (Figure 4C). We removed the ligamentum flavum underneath to allow for a clearer orientation of the neural structures (Figure 4D). Prior to the nerve root becoming visible, the perineural membrane, a surrounding layer or membrane, should be resected first. This action helps clarify the orientation of the root shoulder and axillae, preventing disorientation during surgery (Figure 4E). Then, we remove the ligaments and soft tissue cranial to the root shoulder to make it more easily recognized. The protruding degenerative disc below the nerve root was able to be resected using endo-grasping forceps (Figure 4F). Although a hard disc is commonly encountered in most of these patients and generally does not require removal, we still aim to excise as much of it as possible. The aforementioned procedure constitutes what is known as an ipsilateral cervical foraminotomy. From the images, we can clearly observe the exposure of the ipsilateral spinal cord and nerve root, which have been adequately decompressed. (Figure 4G).

Subsequently, we continued with the use of a large-diameter spinal endoscope, in conjunction with a diamond burr and micro punch, to commence the bony resection at the base of the spinal process, as well as a partial laminectomy of the adjacent cranial and caudal segments on the contralateral side. The removal of the spinal process base is a critical step, as only after adequate removal to create sufficient space can the subsequent endoscopic procedures for contralateral decompression be safely and clearly performed. This includes the use of rongeurs for the removal of contralateral lamina bone or the application of a high-speed burr without the risk of the endoscope inadvertently contacting the spine during contralateral manipulation. (Figure 4H) Following the ample decompression through the removal of bony structures, the contralateral V point becomes clearly visible. (Figure 4I). Afterward, the ligamentum flavum covering the central and contralateral thecal sac was resected with micro-punches. (Figure 4J).

After the substantial removal of the bony base of the spinal process, the contralateral portions of the lamina, and the ligamentum flavum from the midline to the contralateral side, we obtained an adequate operational space for the contralateral foraminotomy. However, before commencing, it is imperative to perform a critical step: the exchange for a finer spinal endoscope. We switched instruments to a standard 30° endoscope with a 6.9-mm outer diameter and a 4.3-mm working channel (FELD scope, Spinendos GmbH, München, Germany). This allowed for a more detailed and deeper contralateral foraminotomy, facilitating the decompression of the opposite lateral recess and foraminal stenosis (Figure 4K). A critical point to note here is that the tip of the protective sheath of the regular spine endoscope must be oriented towards the dorsal side, positioned on the edge of the burred lamina. This ensures the stability of the entire surgical procedure. Similar to the ipsilateral approach, we excised the perineural membrane to visualize the nerve root (Figure 4L). Finally, we continued to utilize the diamond burr for abrasion and cautiously employed micro-punches to partially resect the contralateral superior and inferior articular processes (SAP and IAP) in order to carry out a contralateral foraminotomy. This was performed until the contralateral nerve root was clearly visualized, ensuring complete neural decompression.

### Statical Analysis

SPSS software (Version 26) was used for statistical calculations. Continuous variables were expressed as means with standard error of the mean (SEM) and analyzed using a paired-sample *t*-test to compare repeated measurements. A *p*-value < 0.05 denoted statistical significance.

## 5. Results

**Figure 5:**
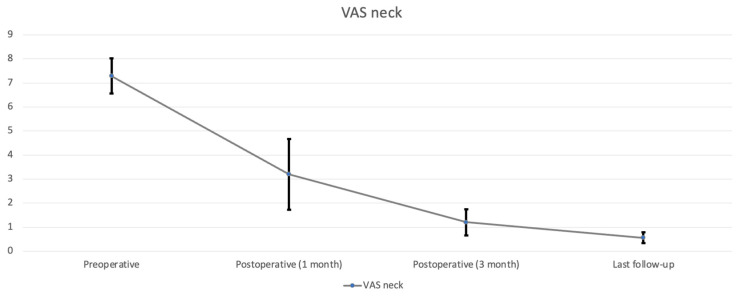
Neck pain was assessed by VAS during follow-up.

**Figure 6:**
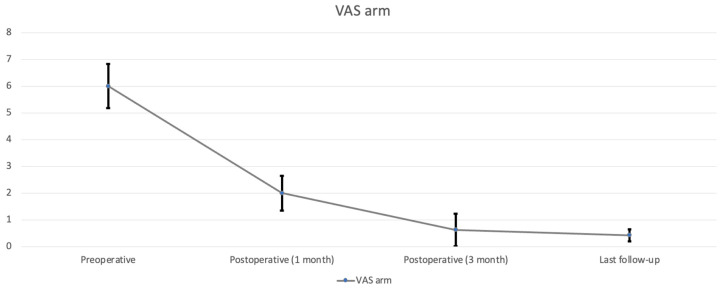
Arm pain assessed by VAS during follow-up.

**Figure 7:**
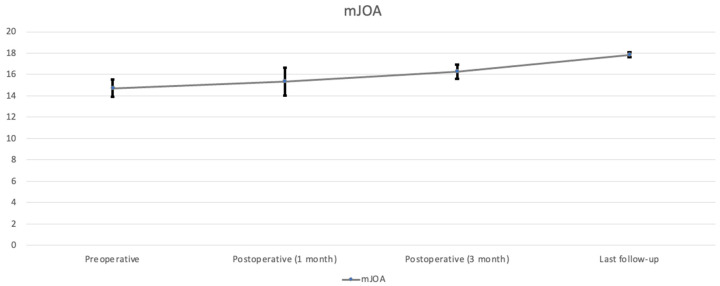
Score of mJOA during follow-up.

All nine patients underwent CE-ULFBD without any major operative complications, such as infection, nerve root injury, or spinal cord damage. The mean follow-up time was 12.1 ± 3.95 months (range, 8–16 months). The mean operation time was 129.56 ± 43.99 min (range, 89–221 min). The mean amount of intraoperative blood loss was 6.56 ± 2.35 mL (range, 5–12 mL). The mean hospital time was 3.44 ± 0.81 days (range 2–5 days). At the last follow-up, the neck pain accessed by VAS decreased from 7.29 ± 0.73 (Table 2, Figure 5) preoperatively to 0.56 ± 0.23 postoperatively (*p* < 0.05), arm pain in VAS decreased from 6 ± 0.82 preoperatively to 0.42 ± 0.23 postoperatively (*p* < 0.05) (Table 2, Figure 6). The score of mJOA improved from 14.71 ± 0.8 preoperatively to 17.83 ± 0.23 postoperatively (*p* < 0.05), and the recovery rate of the mJOA score is 95 percent (Table 2, Figure 7).

#. **Case example 1**

A 75-year-old man reported a two-year history of persistent neck pain, right trapezial pain, bilateral hand pain and numbness, and lower leg weakness. His gait was unsteady, and coordination was impaired, affecting his ability to perform basic tasks such as walking and climbing stairs, and he complained of temporary lower limb weakness. Despite undergoing conservative treatments for 6 months, including physical therapy and pain management, his symptoms persisted and continued to impact his daily life. Consequently, he visited our hospital for further evaluation and surgery.

During the physical examination, the patient exhibited positive results on Spurling’s test, the Shoulder abduction test, and Hoffmann’s test. 

**Figure 8:**
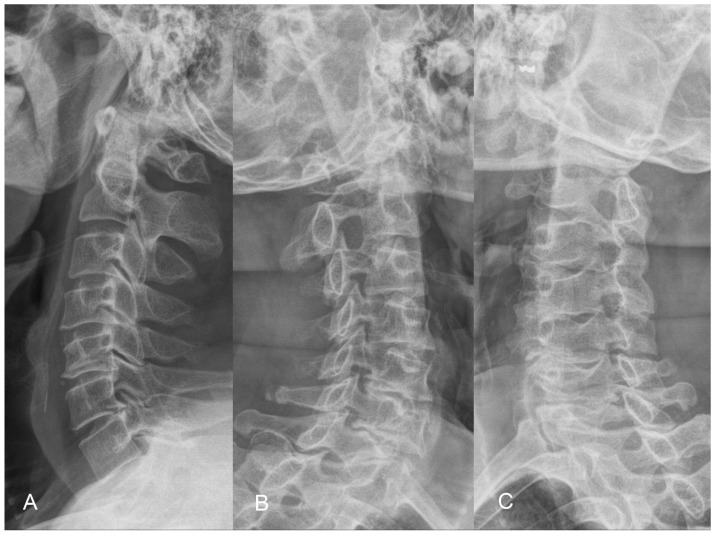
Preoperative lateral (**A**) and oblique radiographs (**B**,**C**) demonstrating well-maintained sagittal lordosis alignment and C3 to C7 foraminal stenosis, accompanied by the formation of osteophytes.

**Figure 9:**
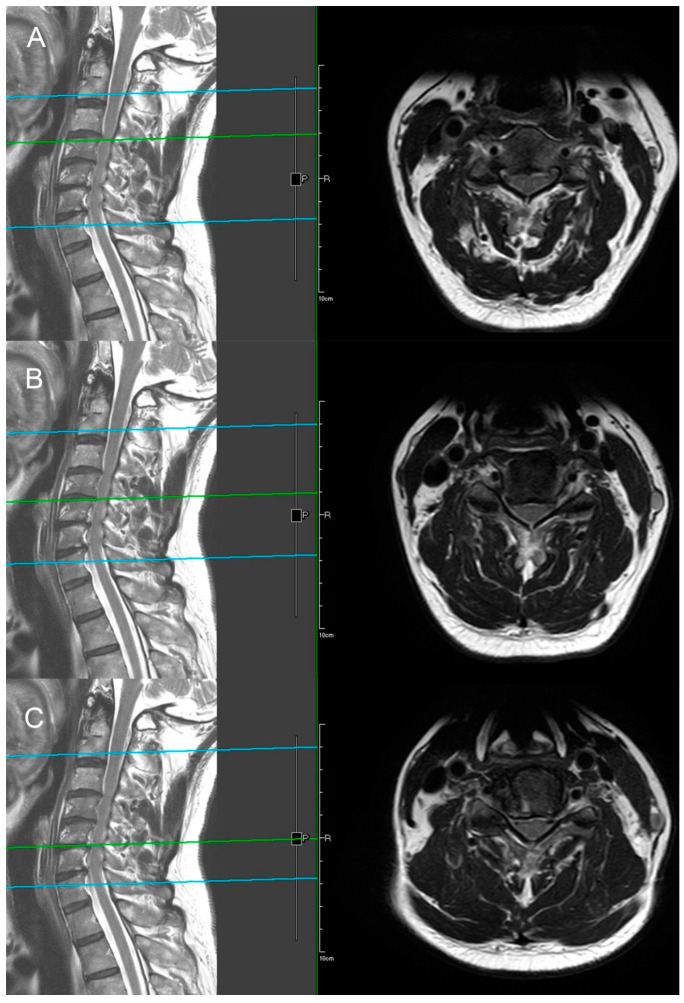
MRI sagittal and axial views illustrating bilateral foraminal stenosis and central stenosis at the C3–C6 levels. ((**A**) C3–C4, (**B**) C4–C5, (**C**) C5–C6). In the left image, the green line represents the relative position of the axial plane MRI of the cervical spine shown in the right image.

The X-ray demonstrates normal cervical lordosis and a positive K-line. There is evidence of bilateral foraminal spur formation at C3-6 and left foraminal spur formation at C6-7. MRI findings indicate C3-7 degenerative spondylosis with central spinal stenosis at C3-6, bilateral foraminal spur formation with stenosis at C3-6, more pronounced on the right side, and left foraminal spur formation with stenosis at C6-7. However, due to the relatively inconspicuous nature of left-sided radiculopathy, surgical intervention at the C6-7 segment was not pursued (Figure 8 and Figure 9). The patient’s preoperative modified Japanese Orthopaedic Association score was recorded as 16, and his visual analog scale neck pain was rated at 8.

**Figure 10:**
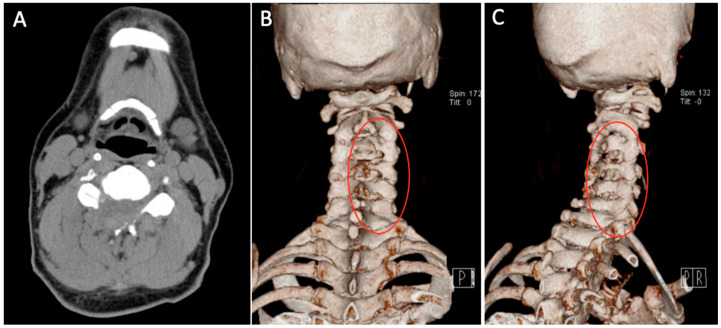
Postoperative axial CT images (**A**) and CT 3-dimensional reconstruction (**B**,**C**). CT = computed tomography. The areas marked by red circles indicate the parts of the cervical spine that were decompressed during surgery.

Patient underwent C3-C6 CE-ULFBD on 8 August 2020. The operation time was 182 min, and blood loss was 12 mL. There were no neurologic complications during operation under the monitoring of MEP (motor-evoked potential) and SSEP (somatosensory-evoked potential). The postoperative radiograph and CT scan revealed successful and substantial decompression of both the foraminotomy and laminotomy procedures (Figure 10). The patients were then discharged on postoperative day 3. At 8-month follow-up, the VAS score for neck discomfort has notably decreased from 7 to 0, while a similar improvement has been noted in the VAS score for hand discomfort, which decreased from 5 to 0. Additionally, there has been a considerable enhancement in lower limb motor strength without temporary lower limb weakness. Furthermore, the modified Japanese Orthopedic Association (mJOA) scores have exhibited positive progress, having risen to 17, reflecting an overall improvement in the patient’s condition.

#. **Case example 2**

This is a case of a 76-year-old female who presented with progressive gait instability nearing one year, which recently exacerbated, significantly impacting daily activities such as walking to the restroom and going for walks, both of which became notably slow and difficult. Additionally, the patient reported bilateral interscapular pain, neck pain, and bilateral upper arm C6 dermatomal pain, which affected her sleep quality. Following ineffective conservative management, including cervical traction, physical therapy, and pain management, she sought consultation at our orthopedic clinic.

Physical examination revealed a positive Spurling Test and Shoulder Abduction Test. Radiographic and MRI imaging demonstrated central canal stenosis compressing the spinal cord from C3 to C6, along with bilateral foraminal narrowing due to osteophytic growth at C5-6, leading to nerve compression.

**Figure 11:**
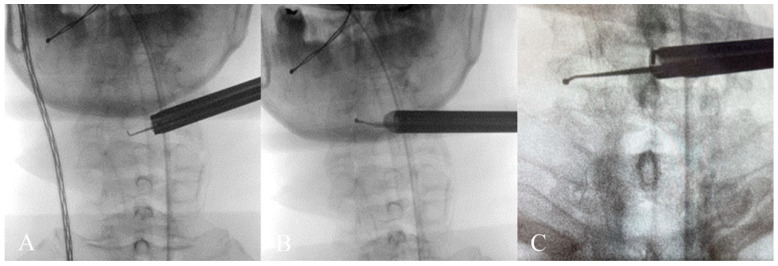
(**A**–**C**) depict intraoperative X-rays during contralateral laminar and foraminotomy decompression, demonstrating the extension of the spinal endoscopic instruments to the far edge of the contralateral foramen.

**Figure 12:**
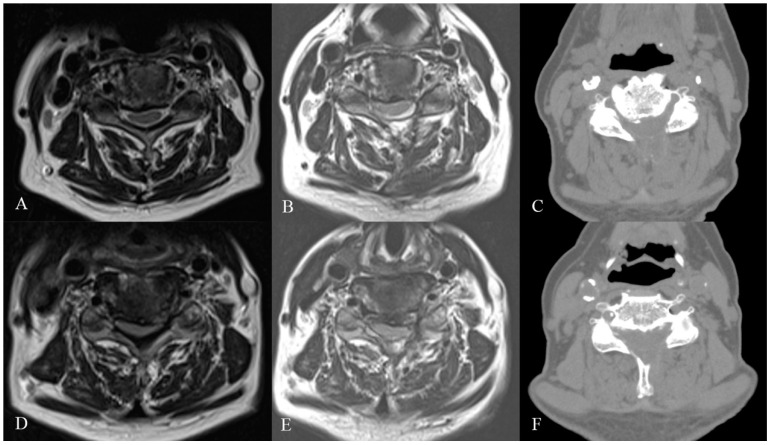
Panels (**A**–**C**) respectively represent preoperative MRI, postoperative MRI, and postoperative CT images of the C4-5 level. Panels (**D**–**F**) correspond to the preoperative MRI, postoperative MRI, and postoperative CT images of the C5-6 level.

The patient underwent C3-C6 CE-ULFBD (Figure 11) in October 2022, with a focus on a thorough bilateral foraminotomy at C5-6 to address the radiculopathy in both upper limbs. The surgery lasted 106 min with a blood loss of 5cc. The patient experienced immediate postoperative pain relief and was discharged the following day to continue recovery at home. The neck pain Visual Analogue Scale (VAS) score improved from 8 preoperatively to 2 postoperatively, and the arm pain VAS score decreased from 8 preoperatively to 1 postoperatively, with both becoming pain-free at six months. Gait instability gradually improved, and the modified Japanese Orthopedic Association (mJOA) score returned to 16 at the one-year follow-up. Figure 12 shows the preoperative and postoperative MRI images.

## 6. Discussion

Although conventional anterior fusion or posterior decompression can relieve neurological symptoms in patients with myeloradiculopathy, their efficacy may be limited by the risk of access-related complications or fusion failure, which can result in the need for revision surgery [13,14,15,16,17,18]. With the introduction of the operating microscope in the 1980s, it allowed surgeons to see the cervical spine more clearly and precisely [19]. The use of retractor or tubular-assisted microscopic cervical decompression reduced the size of the incision and the amount of tissue damage, resulting in shorter hospital stays and faster recovery times compared with traditional open surgery [20]. Recently, high-resolution endoscopes have been developed to see the surgical site with greater clarity and perform more precise surgical maneuvers through targeted approaches. Several clinical studies have compared the clinical outcomes of full-endoscopic technique and microscopic-assisted techniques, and the results show no significant difference between the two groups in terms of clinical success rates and overall complication rates [21,22]. With the benefit of smaller incisions, less tissue damage, scarring, and a faster recovery time, the full endoscopic technique has become a promising alternative option for treating patients with spinal conditions [23].

In 2007, Ruetten et al. introduced the 6.9mm Full-Endoscopic Cervical Posterior Foraminotomy, a keyhole technique that uses a small skin incision of less than 1 cm to access the affected area through a working cannula [23]. The method has been shown to be effective in treating patients with radiculopathy, with a prospective, randomized, controlled study of 87 patients demonstrating positive results compared to traditional surgical methods. However, its use is limited to lateral disk herniation or nerve root canal stenosis, as cervical myelopathy is considered a relative contraindication [23].

In 2019, Y. Lin et al. expanded the scope of Full-Endoscopic Cervical surgery by introducing a posterior percutaneous approach for laminectomy and decompression (PECLD) in treating cervical stenosis with myelopathy caused by hypertrophy of the ligamentum flavum [24]. In a study of 15 patients, he demonstrated the feasibility and safety of the procedure with no reported intraoperative or postoperative complications [24]. In 2020, Dr. Daniel A. Carr made further advancements in the field by translating the concept of unilateral laminotomy for bilateral decompression from the lumbar spine to the cervical spine, providing smaller incisions and better preservation of the posterior osteoligamentous complex [25]. This was achieved by employing a high-speed drill to undercut the base of the rostral spinous process and resecting the bulging contralateral yellow ligament with a Kerrison rongeur [25].

Those muscle-sparing approaches with low complication rates and fast recovery times are typically used in cases of radiculopathy or myelopathy but may not be suitable for all patients with myeloradiculopathy. CE-ULFBD procedure had the advantage of achieving bilateral decompression in patients with myeloradiculopathy through a unilateral posterior approach. For posterior pressure-induced factors like hypertrophic ligamentum flavum, the posterior approach is still an effective approach, and the use of laminotomy instead of laminectomy can also help us preserve spinal stability. By undercutting the base of the spinal process and central lamina, we can not only achieve ipsilateral decompression but also treat contralateral foraminal and spinal stenosis. We employed two types of endoscopes with outer diameters of 10 mm and 6.9 mm, respectively, for surgical procedures. The larger, 10 mm endoscope was primarily utilized for decompression in ipsilateral and central areas. This larger aperture endoscope facilitates more efficient decompression surgeries. Following the partial resection of the base of the spinal process, the created space could accommodate the 6.9 mm smaller scope, enabling intricate contralateral foraminotomy decompression. This surgical approach ensures adequate neural decompression for both the nerve roots and the central spinal cord. Finally, by using the unilateral approach, we can minimize the amount of soft tissue trauma and the incidence of complications to achieve bilateral decompressive spinal surgery in patients with myeloradiculopathy. Results from the latest follow-up in our study show that CE-ULFBD offers improvement as assessed by the VAS and mJOA scores, with no significant complications reported.

In order to perform this technique with familiarity and care, there are several ways that can be facilitated to minimize perioperative complications. The first thing was the prevention of nerve injury [26]. For physicians who are performing this surgery for the first time or are in the early stages of their surgical experience, we recommend the utilization of Motor Evoked Potentials (MEP) and Somatosensory Evoked Potentials (SSEP) to ensure neural safety. These monitoring techniques help in avoiding inadvertent neural damage due to unintentional contact with surgical instruments. Additionally, instead of employing a pumping mechanism, we utilized saline bags suspended not more than 50 cm above the patient. This method is employed to mitigate the risk associated with excessive hydrostatic pressure during the surgical procedure. The surgical team should maintain open communication with the anesthesiologist and inform the patient that the goal is to achieve a systolic blood pressure of less than 110 mmHg to effectively control bleeding. In addition, two units of Tranexamic acid (TXA) can be administered intravenously as a push dose, followed by another two units delivered via IV drip before making the incision to ensure better hemostasis. In our clinical practice, strictly adhering to specific indications when addressing bilateral foraminal stenosis alongside central stenosis is essential for the success of the surgery. Conversely, factors such as cervical instability, abnormal sagittal alignment deformities, tumors, and trauma are considered relative contraindications for this procedure.

Although Endoscopic spinal decompression surgery offers many benefits, it is not without its limitations. Surgeons inexperienced with endoscopic techniques may face a steep learning curve to circumvent perioperative complications such as dural tears, adhesions of the ligamentum flavum, and nerve injuries. Moreover, the relatively lengthy operation time poses a concern that merits attention.

Additionally, this research is constrained by a short follow-up period, and the absence of a control group limits the ability to draw solid conclusions. The long-term effectiveness and potential postoperative complications of this novel surgical technique still warrant comprehensive evaluation. Hence, further investigation and extended monitoring are indispensable to fully elucidate the long-term outcomes and possible complications associated with the CE-ULFBD procedure. 
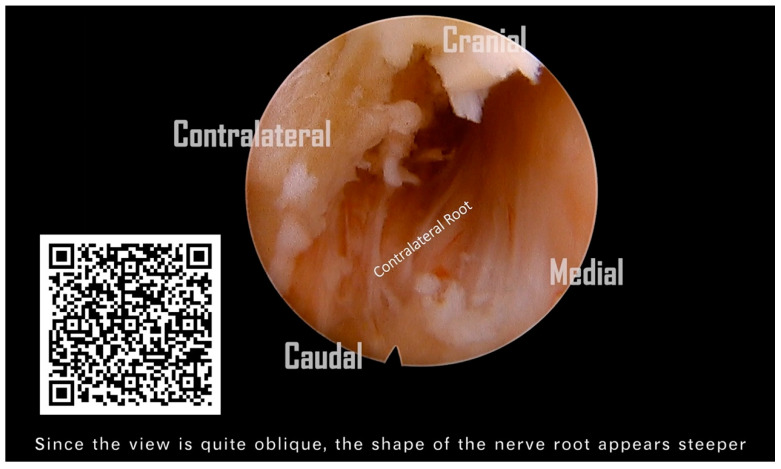


**Video.** https://drive.google.com/file/d/1Ax2-8VqIWfDKkEa6mwVg3pEXs_XxF2rb/view?usp=sharing (accessed on 28 February 2024).

This is a comprehensive demonstration video, accompanied by a 3D schematic diagram in the lower left corner and detailed step-by-step explanations of the procedure.

## Figures and Tables

**Table 1:** Patients characteristics.

Patient No.	Age	Gender	Level	OP Time (Minute)	Blood Loss (mL)	Hospital Stay (Day)
1	74	M	C6–C7	89	5	4
2	75	M	C3–C6	221	12	5
3	72	M	C4–C6	127	7	3
4	68	F	C5–C7	102	5	3
5	62	F	C5–C6	99	7	4
6	76	M	C3–C6	106	5	2
7	63	F	C4–C6	115	5	3
8	66	F	C4–C7	184	8	4
9	69	F	C5–C7	123	5	3

**Table 2:** Comparison of preoperative VAS arm, neck, and mJOA.

	VAS Neck	VAS Arm	mJOA
Preoperative	7.29 ± 0.73	6 ± 0.82	14.71 ± 0.8
Postoperative (1 month)	3.2 ± 1.47	2 ± 0.65	15.32 ± 1.3
Postoperative (3 month)	1.2 ± 0.55	0.62 ± 0.61	16.24 ± 0.66
Last follow-up	0.56 ± 0.23	0.42 ± 0.23	17.83 ± 0.23

## Data Availability

The datasets used and/or analyzed during the current study are available from the corresponding author upon reasonable request.
